# RASAL2 Plays Inconsistent Roles in Different Cancers

**DOI:** 10.3389/fonc.2019.01235

**Published:** 2019-11-13

**Authors:** Bolun Zhou, Wei Zhu, Xingjun Jiang, Caiping Ren

**Affiliations:** Cancer Research Institute, Department of Neurosurgery, Xiangya Hospital, Central South University, Changsha, China

**Keywords:** RASAL2, cancer, EMT, invasion, metastasis, RAS GTPase-activating protein

## Abstract

RAS protein activator like 2 (RASAL2) belongs to the RAS GTPase-activating protein family and plays an important role in several cancers, including ovarian cancer, nasopharyngeal carcinoma, malignant astrocytoma, renal cell carcinoma, bladder cancer, colorectal cancer, liver cancer, triple-negative breast cancer, lung adenocarcinoma, and pancreatic ductal adenocarcinoma. Traditionally, RASAL2 has been regarded as a tumor suppressor but recent studies have found that it is an oncogene in specific types of cancer, such as colorectal cancer, liver cancer, triple-negative breast cancer, triple-negative/estrogen receptor-negative breast cancer. In this review, we summarize the latest findings regarding RASAL2 in cancers, which may be important and useful in clinical practice. We discussed the specific functions and mechanisms of RASAL2 in different kinds of cancer cells (including its inhibition of invasion, metastasis and angiogenesis and its opposite effects), which may provide new directions for cancer research and treatments. RASAL2 exhibits different relationship with clinical cancer stage, histological grade, prognosis and overall survival in different kinds of tumor. RASAL2 is a potential prognostic factor and a new therapeutic target for diagnosis and treatment.

## Introduction

RAS protein activator like 2 (RASAL2) is a member of the family of RAS GTPase-activating proteins (GAP), which negatively regulate the RAS signaling pathway by catalyzing the hydrolysis of RAS-GTP to RAS-GDP. RASAL2 is involved in many cellular activities and acts as a vital regulator of the RAS signaling pathway (1). The RASAL2 gene is located at chromosome 1q25.2 in humans. Early research reported that single-nucleotide polymorphisms (SNPs) located at or near RASAL2 were significantly associated with waist circumference and body mass index in Mexican-Mestizo children and adults (2). Furthermore, the SNP rs10913469 (Sec16B-Rasal2) was positively associated with body mass index in a genome-wide association study (3). RASAL2 promotes adipogenesis through extracellular regulated protein kinases (ERK)-independent repressing RAS activity, which plays a role in the obesity and related metabolic disorders. RASAL2 mutant mice had a drastic decrease in RASAL2 expression, impaired adipogenesis and lean phenotypes (4). Studies have also reported that RASAL2 may play a role in the isolation of cross-reacting antigen (5), creating a candidate vaccine against ticks (6) and lipopolysaccharide-induced activation of microglial cells (7). In addition, the function of RASAL2 can be regulated by microRNAs such as miR-136 (8).

It was reported that RASAL2 acts as a tumor suppressor when it was found that RASAL2 was downregulated in luminal-B breast cancer in one of the earliest studies (9). Recent research reported that RASAL2 suppressed cancer progression via the Hippo signaling pathway (1), WNT/β-catenin pathway (10), and RAS signaling pathway (11). However, RASAL2 also functions as an oncogene in various cancers via the RAS-ERK pathway (12), phosphoinositide 3-kinase (PI3K)/AKT/mechanistic target of rapamycin (mTOR) signaling pathway (9), nuclear factor (NF)-κB pathway (9), and ERK/mitogen activated protein kinase (MAPK) pathway (13). In human cancers, RASAL2 may exhibit pro- or anti-oncogenic behavior depending on the type of stimulus or cell context, which is quite interesting. Thus, what makes RASAL2 thought-provoking is its ability to exert opposite effects in different cancers, which is different from most regulators, which usually exhibit either pro- or anti-oncogenic behavior. The specific mechanisms deserve further study.

## Inhibition of Invasion and Metastasis

**Figure 1:**
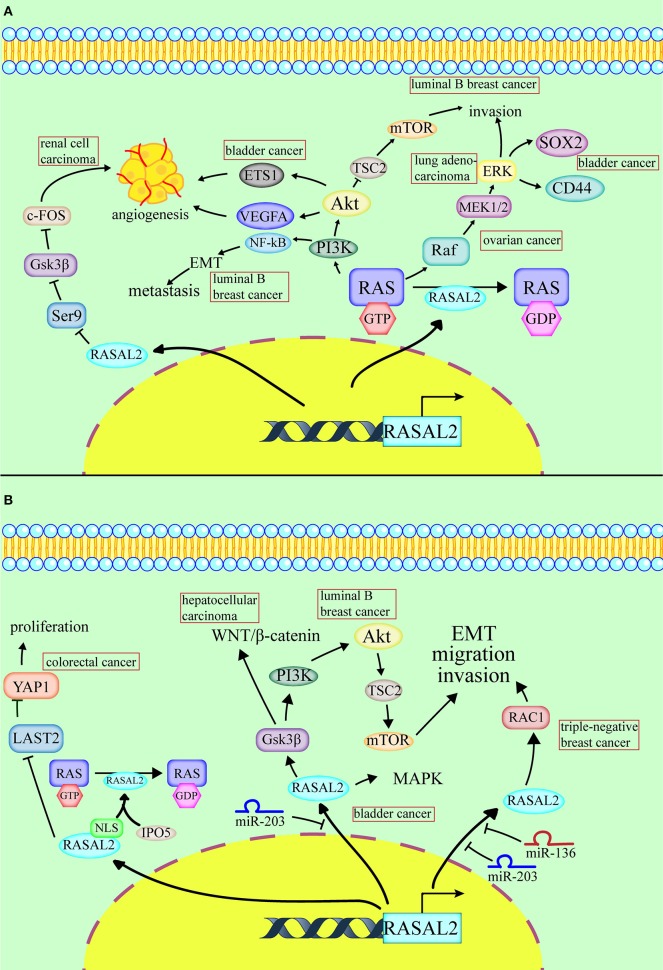
Conflicting roles of RASAL2 in different cancers. **(A)** RASAL2 inhibits angiogenesis, invasion and metastasis of several types of cancer cells. In luminal B breast cancer, the loss of RASAL2 activates wild-type RAS and then increases MEK (mitogen-activated protein kinase kinase)/ERK (extracellular regulated protein kinases) and PI3K (phosphoinositide 3-kinase)/AKT (a serine/threonine-specific protein kinase) signaling to promote invasion, and it induces NF-kB activation to promote epithelial–mesenchymal transition (EMT). In lung adenocarcinoma, the loss of RASAL2 promotes migration capability via EMT through ERK regulation. In ovarian cancer, the loss of RASAL2 activates the RAS-ERK pathway, leading to EMT. In renal cell carcinoma (RCC), RASAL2 reduces Serine9 (Ser9) phosphorylation to activate glycogen synthase kinase-3 β (GSK3β) and then decreases the expression of c-FOS and vascular endothelial growth factor A (VEGFA) to suppress RCC cells. In bladder cancer, RASAL2 depletion enhances the phosphorylation of AKT and then upregulates the expression of ETS proto-oncogene 1 (ETS1) and VEGFA, leading to angiogenesis. RASAL2 also downregulates sex-determining region Y-box 2 (SOX2) and CD44 expression by inhibiting the ERK/mitogen-activated protein kinases (MAPK) pathway to induce cancer stemness. **(B)** RASAL2 promotes invasion and metastasis of several types of cancer cells. In triple-negative breast cancer (TNBC), miR-136 and miR-203 downregulate RASAL2 to suppress cell migration, EMT and invasion. The activation of a RASAL2/ARHGAP24 (Rho GTPase activating protein 24)/RAC1 (RAS-related C3 botulinum toxin substrate 1) module contributes to TNBC tumorigenesis. RASAL2 is hypomethylated and promotes invasiveness in HCC. Downregulation of RASAL2 alters the phosphorylation of the effectors of the MAPK, WNT/β-catenin, and PI3K/AKT/mTOR signaling pathways and then impairs these pathways. RASAL2 is also the target of miR-203 in HCC cells. In colorectal cancer (CRC), importin-5 (IPO5) binds to the nuclear localization signal or sequence (NLS) sequence of RASAL2, which induces RAS signal activation. RASAL2 is also involved in the Hippo signaling pathway, which promotes tumorigenesis and metastasis by inhibiting the expression of large tumor suppressor kinase 2 (LATS2) and then increasing the expression of yes-associated protein 1 (YAP1) in CRC.

The RAS pathway is one of the most commonly deregulated pathways in human cancer (14). RASAL2 plays an important role in the invasion and metastasis of some cancer cells, which regulates the action of the cancer cells (Table 1, Figure 1A). The RAS-GAP domain of RASAL2 is important for the activity of RASAL2. That RAS protein contribute to the oncogenesis caused by RASAL2 inactivation (9). In most cancers, RASAL2 is inhibited and downregulated so that the cancer cells can escape from its preventative effects.

**Table 1:** Different RASAL2 functions in different cancers.

**Cancer type**	**Expression**	**Role**	**Regulation**	**Function**	**References**
Lung adenocarcinoma	Downregulated	Suppressor	↓RASAL2/↑EMT	Inhibiting lymph node metastasis, invasion, migration, metastasis	(15)
Ovarian cancer	Downregulated	Suppressor	↓RASAL2/↑RAS-ERK pathway/↑EMT	Negatively associated with pathological grade, inhibiting proliferation, migration, invasion	(12)
Pancreatic ductal adenocarcinoma	Downregulated	Suppressor	↑sulforaphane/↑miR135b-5p/↑RASAL2/↓ERK1/2	Inhibiting cancer progression, malignancy	(16)
Nasopharyngeal carcinoma	Downregulated	Suppressor	↓RASAL2/↑EMT	Inhibiting proliferation, invasion, migration, metastasis	(17)
Malignant astrocytoma	Downregulated	Suppressor	↑ECT2/↓RASAL2/↑MAT	Inhibiting invasion, migration	(18)
Renal cell carcinoma	Downregulated	Suppressor	DNA promoter methylation/↓RASAL2/↑p-GSK3/↑c-FOS/↑VEGFA	Negatively associated with tumor stage and grade, inhibiting proliferation, angiogenesis	(19)
Bladder cancer	Downregulated	Suppressor	↓RASAL2/↑p-ERK/↑SOX2 ↓RASAL2/↑p-AKT/↑ETS1/↑VEGFA	Inhibiting cancer recurrence, stemness, metastasis Inhibiting metastasis, angiogenesis	(13, 20)
Luminal B breast cancer	Downregulated	Suppressor	↓RASAL2/↑RAS-ERK pathway	Inhibiting migration, invasion, cancer progression, metastasis	(9, 21–23)
Liver cancer	Lower than adjacent tissue	Suppressor	↓RASAL2/↓recurrence-free survival and overall survival	Positively associated with degree of differentiation, TNM stage, inhibiting vascular invasion	(24)
Colorectal cancer	Downregulated	Suppressor	↓RASAL2/↑p-ERK/↑EMT	Inhibiting proliferation, invasion, migration, metastasis	(25)
Colorectal cancer	Unclear	Suppressor	↑IPO5/↑RASAL2 nuclear transportation/↑RAS signal activation	Inhibiting proliferation, migration, metastasis	(11)
Colorectal cancer	Upregulated	Oncogene	↑RASAL2/↓LATS2/↑YAP1	Proliferation, metastasis, oncogenesis	(1)
Liver cancer	Upregulated	Oncogene	↓RASAL2/↓RSK1/↓RSK2/↓p-JNK/↓p-MKK6 ↑miR-203/↓RASAL2/↓p-AKT	Proliferation, invasion, metastasis Proliferation, invasion	(10, 26)
Triple-negative breast cancer	Upregulated	Oncogene	↑miR-136 /↓RASAL2/↓EMT	Invasion and metastasis, positively associated with pathological grade	(8)
Triple-negative/estrogen receptor-negative breast cancer	Upregulated	Oncogene	↓miR-203/↑RASAL2/↑RAC1	Poor clinical outcomes, early metastasis, disease recurrence, oncogenesis, mesenchymal cell invasion	(27)

*RASAL2 is downregulated in most cancers and functions as a suppressor. However, RASAL2 is also reportedly overexpressed in some cancers. This table presents the specific mechanism, expression and function of RASAL2 in different cancer types. EMT, epithelial–mesenchymal transition; ERK, extracellular regulated protein kinases; ECT2, epithelial cell transforming factor 2; MAT, mesenchymal–amoeboid transition; VEGFA, vascular endothelial growth factor A; AKT, a serine/threonine-specific protein kinase; SOX2, sex-determining region Y-box 2; GSK3, glycogen synthase kinase-3, ETS1, ETS proto-oncogene 1; ERK, extracellular regulated protein kinases; YAP1, yes-associated protein 1; RSK1, ribosomal S6 kinase 1; RSK2, ribosomal S6 kinase 2; RAC1, RAS-related C3 botulinum toxin substrate 1*.

The most common cancer that is associated with RASAL2 is breast cancer, such as luminal B breast cancer. In luminal B breast cancer, due to promoter hypermethylation, disabled homolog 2-interacting protein (DAB2IP) is selectively suppressed (21). The combined loss of RASAL2 and DAB2IP in a subset of luminal B breast cancers results in epithelial–mesenchymal transition (EMT). In a luminal B mammary tumor mouse model, the loss of RASAL2 enhanced metastasis and, using this model, 60% of primary tumors spontaneously lost DAB2IP, which also increased metastasis. As for the mechanism, the loss of DAB2IP and RASAL2 increased MEK/ERK and PI3K/AKT signaling (which are quite important for invasion) and induced NF-κB activation to promote EMT (21).

RASAL2 negatively regulates EMT via the ERK pathway. For example, in lung adenocarcinoma, downregulation of RASAL2 promotes migration capability due to EMT via ERK regulation (15). Thus, RASAL2 may be important in lung adenocarcinoma treatment (15). In ovarian cancer, RASAL2 was downregulated, especially in patients with advanced stages and grades. Downregulation of RASAL2 activated the RAS-ERK pathway, leading to EMT, and inhibition of the pathway reversed the functional effects of RASAL2 depletion (12).

MiR135b-5p is induced by sulforaphane and then promotes the expression of RASAL2 to suppress pancreatic ductal adenocarcinoma (16). Low expression of miR-135b-5p and RASAL2 are indicators of pancreatic cancer malignancy (16). RASAL2 also inhibited the metastasis capability and proliferation of nasopharyngeal carcinoma (17). In astrocytoma cells, RASAL2 interacts with epithelial cell transforming factor 2 (ECT2), which activates Rho GTPases, in order to reduce Rho activity. When the ratio of ECT2 to RASAL2 activity is increased, ECT2 overcomes the RhoGAP activity of RASAL2 and results in mesenchymal–amoeboid transition (MAT), resulting in amoeboid cells (18).

## Inhibition of Angiogenesis

RASAL2 plays a critical role in inhibiting tumor angiogenesis, which means that it can act as a tumor suppressor (Table 1, Figure 1A). In renal cell carcinoma (RCC), RASAL2 is usually epigenetically silenced and its loss is negatively associated with overall survival of RCC patients. RASAL2 targets tumor angiogenesis to suppress RCC cells. Mechanistically, RASAL2 reduces Ser9 phosphorylation to activate glycogen synthase kinase-3 β (GSK3β) and subsequently downregulates the expression of c-FOS and vascular endothelial growth factor A (VEGFA) (19).

In bladder cancer, RASAL2 is downregulated and negatively associated with clinical stage. RASAL2 inhibits angiogenesis of cancer cells via p-AKT/ETS proto-oncogene 1 (ETS1) signaling. Mechanistically, RASAL2 depletion enhances the phosphorylation of AKT and subsequently upregulates the expression of ETS1 (an important angiogenesis-related protein) and VEGFA. There is a negative association between RASAL2 and VEGFA expression, showing that RASAL2 inhibits angiogenesis via regulating VEGFA (20). RASAL2 also downregulates sex-determining region Y-box 2 (SOX2) and CD44, which are indicators of cancer stemness, by inhibiting the ERK/MAPK pathway (13).

## Promotion of Invasion and Metastasis

RASAL2 plays inconsistent roles in different cancers, including promoting invasion and metastasis of some cancer cells and not others (Table 1, Figure 1B). RASAL2 is a target of anti-invasion miR-136 and miR-203 in triple-negative breast cancer (TNBC). RASAL2 drives mesenchymal invasion and metastasis and miR-136 and miR-203 downregulate RASAL2 to suppress cell migration, EMT and invasion (8). Furthermore, high RASAL2 expression is strongly associated with poor disease outcomes in TNBC patients. As for the mechanism, RASAL2 binds and antagonizes the RAS-related C3 botulinum toxin substrate 1 (RAC1)-specific Rho GTPase activating protein 24 (ARHGAP24) to promote signaling involving the small GTPase RAC1 and thereby promote mesenchymal invasion. To summarize, the activation of a RASAL2/ARHGAP24/RAC1 module contributes to TNBC tumorigenesis (27).

In hepatocellular carcinoma (HCC), RASAL2 is hypomethylated and RASAL2 is upregulated, thereby promoting invasiveness. Downregulation of RASAL2 alters the phosphorylation of the effectors of the MAPK, WNT/β-catenin and PI3K/AKT/mTOR signaling pathways and then impairs these pathways. For instance, RASAL2 depletion impairs phosphorylation of ribosomal S6 kinase 1 (RSK1) and ribosomal S6 kinase 2 (RSK2), which are downstream effectors of both PI3K and MAPK. Phosphorylation of GSK3β reduces its enzymatic activity and induces the WNT/β-catenin pathway (28). RASAL2 depletion attenuated GSK3β phosphorylation, which decreases AKT and RSK1/2 activities and downregulates WNT signal transduction (10). RASAL2 is also the target of miR-203 in HCC cells (26).

In colorectal cancer (CRC), RASAL2 is overexpressed and RASAL2 nuclear transportation is mediated by importin-5 (IPO5) to promote proliferation and tumorigenicity (25). IPO5 binds to the nuclear localization signal or sequence (NLS) of RASAL2, which induces RAS signal activation (11). RASAL2 is also involved in the Hippo signaling pathway, which promotes tumorigenesis and metastasis by inhibiting the expression of large tumor suppressor kinase 2 (LATS2), which increases the expression of yes-associated protein 1 (YAP1) in CRC. YAP1 is dephosphorylated and translocated to the cell nucleus, which promotes the expression of pro-proliferation genes and prevents YAP1 ubiquitination in the cytoplasm (1). The recent findings on RASAL2, which functions as a tumor suppressor or oncogene in different cancers, are presented in Table 1.

## Conclusions and Future Perspectives

RASAL2 functions as a tumor suppressor and inhibits cancer progression in lung cancer (15), ovarian cancer (12), pancreatic ductal adenocarcinoma (16), nasopharyngeal carcinoma (17), malignant astrocytoma (18), RCC (19), bladder cancer (13, 20), and luminal B breast cancer (9, 21, 23). Thus, downregulation of RASAL2 often exhibits a positive relationship with clinical cancer stage and histological grade. In addition, low expression of RASAL2 often indicates a poor prognosis and is associated with overall survival. Following RASAL2 suppression, the RAS-ERK pathway is activated and functions as a promoting factor. Therefore, blockade of the RAS-ERK pathway using inhibitors may be effective in the treatment of cancers involving RAS pathway activation. Additionally, restoring RASAL2 expression or synthesizing a small molecule that can replace RASAL2 may be a novel approach to cancer treatment. Briefly, RASAL2 is a potential prognostic factor and a new therapeutic target for cancer diagnosis and treatment.

However, several studies have reported that RASAL2 was oncogenic in TNBC (8, 27), HCC (10, 26), and CRC (1). Overexpression of RASAL2 may be a predictive and prognostic marker in TNBC, as it is positively associated with clinical cancer stage and histological grade. These opposing conclusions may be the results of functional complexity, cancer microenvironments and the heterogeneity of molecular cancer pathways. The specific mechanisms deserve further study.

## Author Contributions

BZ and WZ designed and wrote the manuscript. XJ and CR designed and revised the manuscript.

### Conflict of Interest

The authors declare that the research was conducted in the absence of any commercial or financial relationships that could be construed as a potential conflict of interest.
